# Differential Immune Transcriptome and Modulated Signalling Pathways in Rainbow Trout Infected with Viral Haemorrhagic Septicaemia Virus (VHSV) and Its Derivative Non-Virion (NV) Gene Deleted

**DOI:** 10.3390/vaccines8010058

**Published:** 2020-01-30

**Authors:** Blanca Chinchilla, Paloma Encinas, Julio M. Coll, Eduardo Gomez-Casado

**Affiliations:** 1 Ocular Genomics Institute, Department of Ophthalmology, Massachusetts Eye and Ear Infirmary and Harvard Medical School, Boston, MA 02114, USA; Blanca_chinchillarodriguez@meei.harvard.edu; 2 Department of Biotechnology, National Agricultural and Food Research and Technology Institute (INIA), 28040 Madrid, Spain; paloma.encinas@inia.es (P.E.); juliocoll@inia.es (J.M.C.)

**Keywords:** VHSV, non-virion (NV), transcriptome profiling, rainbow trout, immune pathways

## Abstract

Viral haemorrhagic septicaemia virus (VHSV) is one of the worst viral threats to fish farming. Non-virion (NV) gene-deleted VHSV (dNV-VHSV) has been postulated as an attenuated virus, because the absence of the *NV* gene leads to lower induced pathogenicity. However, little is known about the immune responses driven by dNV-VHSV and the wild-type (wt)-VHSV in the context of infection. Here, we obtained the immune transcriptome profiling in trout infected with dNV-VHSV and wt-VHSV and the pathways involved in immune responses. As general results, dNV-VHSV upregulated more trout immune genes than wt-VHSV (65.6% vs 45.7%, respectively), whereas wt-VHSV maintained more non-regulated genes than dNV-VHSV (45.7% vs 14.6%, respectively). The modulated pathways analysis (Gene-Set Enrichment Analysis, GSEA) showed that, when compared to wt-VHSV infected trout, the dNV-VHSV infected trout upregulated signalling pathways (*n* = 19) such as RIG-I (retinoic acid-inducible gene-I) like receptor signalling, Toll-like receptor signalling, type II interferon signalling, and nuclear factor kappa B (NF-kappa B) signalling, among others. The results from individual genes and GSEA demonstrated that wt-VHSV impaired the activation at short stages of infection of pro-inflammatory, antiviral, proliferation, and apoptosis pathways, delaying innate humoral response and cellular crosstalk, whereas dNV-VHSV promoted the opposite effects. Therefore, these results might support future studies on using dNV-VHSV as a potential live vaccine.

## 1. Introduction

Viral haemorrhagic septicaemia virus (VHSV) belongs to the *Novirhabdovirus* genus, together with infectious haematopoietic necrosis virus (IHNV), snakehead rhabdovirus (SHRV), and hirame rhabdovirus (HIRV). They are all enveloped negative-stranded RNA viruses with a single RNA genome of ~11 Kb [1,2,3], which encodes five virion proteins (N, P, M, G, and L proteins) and the non-virion (NV) protein that gives the name to the *Novirhabdovirus* genus and differentiates it from other fish rhabdoviruses such as spring viremia carp virus (SVCV). VHSV has been isolated from more than 50 fish species from North America, Asia, and Europe, including 15 farmed [4] and free-living marine fish species [5] like trout, salmon, turbot, and eel, among others. Within a farm, the presence of VHSV infection, even if in only one individual fish, has to be notified to the Office International des Epizooties (OIE, Paris, France) and implies the sacrifice of all the farmed fish, thus leading to serious economic losses [6,7]. The *NV* gene was firstly characterised and named from IHNV genome studies [8]. Some years later, the *NV* gene from VHSV was further characterised by comparative genome studies [9]. Despite the presence of the *NV* gene in the four novirhabdovirus species mentioned above, their NV proteins showed very divergent inter-species sequences [10,11]. Initial studies regarding NV role showed that it was required for the highest efficient replication of IHNV in rainbow trout [12,13,14] and that of VHSV in olive flounder [13,15] and in *Epithelioma papulosum cyprinid* (EPC) cells [13]. However, NV was not essential for in vitro or in vivo SHRV production in warm-water flatfish [16,17]. Further, in vitro studies using the wild-type (wt) and NV knock-out IHNV or VHSV suggested that NV downregulated the host *ifn1*/*mx* transcriptional levels during in vitro infection in trout (RTG-2, Rainbow Trout Gonad-2) [18] or EPC cells [15], respectively. The higher levels of IFN-induced *mx* transcript in NV knock-out VHSV vs. wt-VHSV injected flounder found in these studies suggested that NV also interferes with IFN defences in vivo to favour VHSV replication [15]. The early anti-apoptotic role of NV during the first stages of VHSV infection has been also demonstrated [19]. Using recombinant NV protein (rNV) and a trout immune-targeted microarray, we have previously determined not only an anti-apoptotic role for NV, but also a plethora of novel expression changes (mainly downregulated) in genes associated with immune innate and adaptive response (i.e., interferons, MX, tumour necrosis factors, antigen presentation, interleukins) [6]. However, the effects driven by the injection of the rNV protein alone will probably differ from those induced by the NV in the course of VHSV infection. Recently, a microarray study in olive flounder liver infected with VHSV described differential gene expression and gene ontology classification of these genes [20], resulting in a global transcriptome profiling where only a few genes have been classified as immune-related. Gene expression has been also characterised with microarrays in olive flounder infected with a VHSV strain that produces high mortality in this species [21]. In addition, the protection of olive flounder against VHSV was previously assessed by immunization with the *NV* gene-knockout recombinant VHSV, which led to good protection against virulent VHSV [22]. However, the underlying mechanisms of olive flounder protection remain unknown. The aim of this work was to characterise the transcriptomic profiling of immune-related genes in trout infected with the wild-type VHSV (expressing NV) and dNV-VHSV (*NV* gene-deleted) in order to find targeted immune genes and signalling pathways implied in the course of a VHSV infection. This study will contribute to better understand how NV modulates gene expression and how the expression pattern changes in response to dNV-VHSV. The results will help tailoring future vaccines against viral haemorrhagic septicaemia virus and other novirhabdoviruses.

### 1.1. Viral Haemorrhagic Septicaemia Viruses (VHSV)

Wild type VHSV-23.75 (wt-VHSV) isolated from brown trout [23] (GenBank accession number FN665788) and its derivative *NV* gene-deleted (dNV-VHSV), obtained as previously described [13], were used to infect rainbow trout (*Oncorhynchus mykiss*) by intraperitoneal injection. Both wt- and dNV-VHSV viruses were kindly provided by Dr. Michel Brémont (INRA, France), and further propagated in EPC cells at 14 °C and titrated by the plaque-forming assay (pfu) as previously described [14].

### 1.2. Virus Dosages and Injection of Fingerling Rainbow Trout

Fingerling rainbow trout (*Oncorhynchus mykiss*) of 6–12 g (approximately 10 cm in length), free of IPNV (Infectious Pancreatic Necrosis Virus) and VHSV antibodies, were obtained from a local fish farm (Los Molinos, Madrid). They were maintained at 14 °C in a 200 L aquarium with tap-dechlorinated carbon-filtered water provided with biological filters and fed with a commercial fish diet. After two weeks of acclimation, fish were separated into seven groups of six trout per group. Due to the non-virion (NV) expression by wt-VHSV, a different replication rate of wt-VHSV and dNV-VHSV in the EPC cell line was established [13], and since the maximum NV expression by wt-VHSV is reached at 48 hpi (hours post-infection), we aimed at establishing the equivalent infectious dosage yielding comparable transcriptomic profile for each virus at 48 hpi. For that, we injected trout intraperitoneally with 100 µL of wt-VHSV (10^4^, 10^5^ or 35 × 10^6^ pfu), dNV-VHSV (10^4^, 10^5^ or 35 × 10^6^ pfu) or phosphate-buffered saline (PBS). Each group of injected trout was then released into a 50-L aquarium and maintained at 14 °C. Two days after injection, trout were sacrificed, head kidney and spleen (whole organs) were pooled, and immediately immersed in RNAlater (Ambion, Austin, USA) at 4 °C overnight, before being frozen at −70 °C until further analysis.

### 1.3. RNA Extraction and cDNA Synthesis

The pooled head kidney and spleen (whole organs) from each individual trout were homogenized using the Tissue Lyser Cell Disruptor (Qiagen Iberia, S.L., Madrid, Spain) for 10 min at 50 Hz with 3 mm glass beads in an RTL buffer (Qiagen Iberia, S.L., Madrid, Spain). RNA was then extracted from the homogenates by using the RNAeasyPlus kit (Qiagen Iberia, S.L., Madrid, Spain) and eluted in RNase-free water. RNA concentrations were estimated by Nanodrop and the presence of 18S and 28S rRNA bands was confirmed by denaturing RNA agarose electrophoresis (Sigma-Aldrich Quimica SA, Madrid, Spain). For qPCR experiments of the nucleoprotein (*N*) and non-virion (*NV*) genes, cDNA synthesis was carried out from RNA (1 µg) by using oligo-dT and PrimeScript^TM^ reverse transcriptase (RR037A TAKARA, Japan) according to the manufacturer’s instructions. For microarray experiments, additional RNA quality controls (RNA integrity number, RIN) were performed by NIMGenetics (Madrid, Spain). For each experimental six-trout group, the four trout with best RNA quality (RIN > 7.0) were chosen for microarray hybridisation. cDNA was synthesized by using SuperScript III reverse transcriptase (Invitrogen) and oligo(dT) primer, labelled with Cy3 (GE Healthcare, Spain), and purified with Microcon YM30 (Merck Millipore, Spain).

### 1.4. Design of Oligo-Microarrays Enriched in Rainbow Trout Immune-Related Genes (Targeted Microarrays)

Oligo-microarrays were enriched in rainbow trout immune-related genes as previously described (immune-targeted microarrays) [6,24,25]. The final 8 × 15K microarray corresponds to Agilent’s ID032303 (Gene Expression Omnibus GEO platform submission number GPL14155) and contains 1474 annotated immune-related probes (60-mer) per duplicate. In order to simplify the analysis of results, annotated probes were classified according to the following gene groups: VIG, VHSV-induced genes (number of probes, *n* = 22); IFN, interferons and their receptors (*n* = 20); MX, interferon-inducible proteins mx (*n* = 3); CO, complement components (*n* = 6); IL, interleukins and their receptors (*n* = 19); APM, antigen-presenting machinery genes (*n* = 4); TNFSF, tumour necrosis factor superfamily (*n* = 16); CD, cluster differentiation antigens (*n* = 15); CK, chemokines and their receptors (*n* = 32); CASP, caspases (*n* = 3); and TF, transcription factors (*n* = 10). The trout microarray used for these experiments was previously validated by real-time quantitative PCR (RTqPCR) [24,25]. The number of biological replicas was four. Four chips of eight samples per chip were used and hybridised simultaneously. This home-made rainbow trout oligo-microarray contains more immune-related genes than any other trout microarray available, since it includes all the immune-related genes from the Agilent’s EST-derived rainbow trout oligo-microarray (ID16271) [6,24,25,26].

### 1.5. Hybridisation and Gene Expression Changes of Trout Transcripts to the Immune-Targeted Microarrays

The labelling of 2 µg of RNA (approximately 50 µg/mL) and hybridisation to the microarrays were performed by NIMGenetics (Madrid, Spain) complying with the Minimum Information about a Microarray Experiment (MIAME) standards [24].

Normalisations were performed by correcting the individual fluorescence in each microarray with the sum of all the fluorescent values according to the formula: fluorescence of each probe/sum of all the probe fluorescence signals per microarray. Raw and normalised data were deposited in GEO [27,28]. After normalisation, outlier values (defined by those fluorescence values above or below mean ± standard deviation per probe) were identified and eliminated from the calculations programmed in Origin Pro 8.6 (OriginLab Corporation, Northampton, MA, USA). Fold-change (FC) for each probe was calculated by the following formula: values of wt-VHSV or dNV-VHSV injected trout/mean of PBS injected trout (*n* = 4). Means and standard deviations of individual folds were calculated for each oligonucleotide probe by the following formula: fluorescent value/mean fluorescent value of the control (*n* = 4). Venn diagrams reflect the percentage of genes which FC value was upregulated, downregulated, and non-regulated for each comparison with these arbitrary criteria previously used [6]: (1) upregulated: FC ≥ 1.5; (2) downregulated: FC ≤ −1.5; and (3) non-regulated (basal) gene expression: −1.5 < FC < 1.5. On the other hand, the heatmap figures reflect the FC for each gene comparison using an arbitrary criteria previously described [6]: (1) non-regulated (basal) gene expression: −1.5 < FC < 1.5 (black); (2) upregulated: 1.5 ≤ FC < 2 (light red box), 2 ≤ FC < 5 (red box), 5 ≤ FC (dark red box); and (3) downregulated: –1.5 ≥ FC > −2 (light green box), −2 ≥ FC > –5 (green box), −5 ≥ FC (dark green box). Differentially expressed gene transcripts were considered significant when FC ≥ 1.5 or FC ≤ −1.5. Negative folds were calculated for those values below 0.66 applying the formula −1/FC. Therefore, FC = 0.66 corresponds to a –1.5 value; FC = 0.5 (more downregulated) corresponds to a −2 value; and FC = 0.2 (even more downregulated) corresponds to a –5 value.

### 1.6. Quantitative Estimation of Transcripts by Real-Time Quantitative PCR (RTqPCR)

To estimate the wt- or dNV-VHSV replication in rainbow trout head kidney and spleen, both *N* and *NV* transcript levels were estimated by RTqPCR amplification after intraperitoneal injection of the corresponding VHSV, as described in Section 1.2. RNA extraction and cDNA synthesis were carried out as described above. RTqPCR was carried out by mixing 100 ng of cDNA, 0.9 μM of forward primer, 0.9 μM of reverse primer, and Power SYBR Green PCR Master Mix (Life Technologies, Madrid, Spain). The thermal profile was 10 min at 95 °C, followed by 40 cycles of 95 °C for 15 s, and 60 °C for 1 min. For each experiment, the expression level of the analysed genes was calculated using the 2^-ΔΔCt^ relative quantitation method. The Ct for each viral gene was normalised to *β-actin* gene (∆Ct*^gene^* = Ct*^gene^* − Ct*^β-actin^*), which was used as an internal control due to its low coefficient of variation (CV) among different virus dosages (CV ≤ 3% for 10^4^ pfu/trout and 10^5^ pfu/trout of wt- and dNV-VHSV; trout injected with 35 × 10^6^ pfu of both viruses showed a CV close to 8%). Means and standard deviations were calculated for each experimental infection by intraperitoneal (ip) injection of either the wt- or dNV-VHSV in trout groups (*n* = 6) with 10^4^, 10^5^, or 35 × 10^6^ pfu/trout. Primer sequences used were: *β-actin* (accession number AF550583.1) forward 5′CATCACCATCGGCAACGA and reverse 5′GATGTCCACGTCACACTTCAT; nucleoprotein (accession number AJ233396) forward 5′TCTCCGCTCGTCCTCCGTGAG and reverse 5′GTGAGCCCAGAGCCTCTTGTC; and non-virion (*NV*, accession number AJ233396) forward 5′TCAAGGTGACACAGGCAGTCA and reverse 5′CCAGTTCTCTCATGGGCATCAT. Calculated RTqPCR efficiency was 59% for *β-actin*, 45% for *N*, and 43% for *NV* genes. Efficiency was considered to correct the transcript levels obtained by RTqPCR assays.

### 1.7. Calculations used for Gene Set Enrichment Analysis (GSEA)

In order to explore the possible biological effects of simultaneous and small changes in several related genes, we screened the transcriptional data with the previously described 51 rainbow trout from the immune-related gene-set (GS) collection [25]. The trout GS collection was manually designed from the KEGG (K) and WIKI (W) trout orthologous human pathways (as accessed in 2013), using the trout genes contained in our home-designed microarray. The trout GS collection was then used for analysis by the Gene-Set Enrichment Analysis (GSEA) program [29,30,31]. Transcriptional data from the dNV-VHSV and wt-VHSV injected trout were analysed by GSEA to assign a normalised enrichment score (NES) for each GS of the collection in each of the three cases [25].

### 1.8. Ethics Statement

All the animal procedures used in this study were approved by the INIA (National Agricultural and Food Research and Technology Institute) ethical and biosecurity committee (authorization CEEA 2011/022) and performed following the National and European Commission guidelines and regulations on laboratory animals’ care. Periodic examinations were performed several times a day during infections so as to euthanize fish with abnormal behaviour. To minimize animal suffering, fingerling rainbow trout were sacrificed by using a lethal dose of tricaine methanesulfonate (MS-222, 50 mg/mL, Sigma, Madrid, Spain).

## 2. Results and Discussion

### 2.1. dNV- and wt-VHSV Dosages used for Microarray Analysis

**Figure 1:**
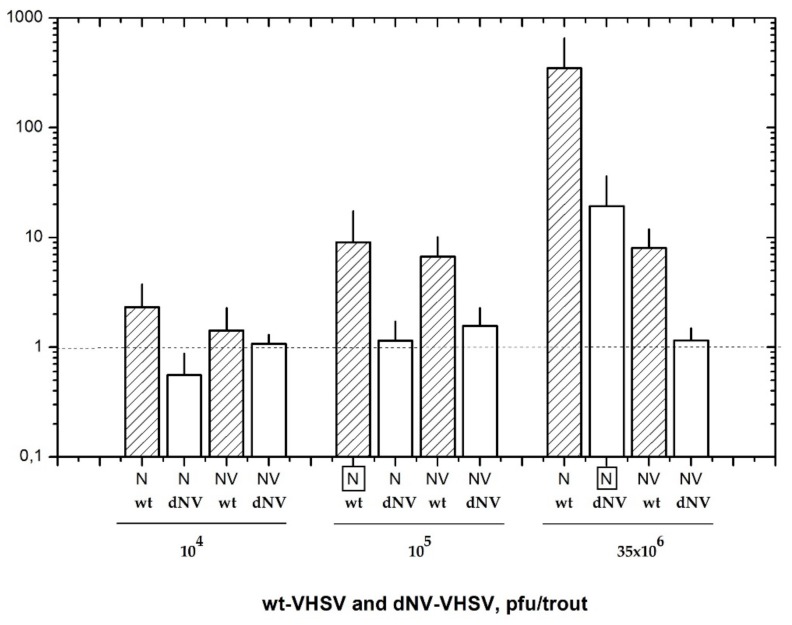
Nucleoprotein (*N*) and non-virion (*NV*) gene expression values (mean ± SD) obtained by RTqPCR of six trout injected with wild-type (wt)-VHSV (hatched bars) and *NV* gene-deleted (dNV)-VHSV (white bars). Animals were injected with three different dosages (10^4^, 10^5^, and 35 × 10^6^ pfu/trout) of each virus and analysed 48 hpi. The results showed similar nucleoprotein (boxed N) transcript levels of wt-VHSV (10^5^ pfu/trout) and dNV-VHSV (35 × 10^6^ pfu/trout), indicating their similar replication levels. Horizontal dotted line corresponds to negative control. VHSV: viral haemorrhagic septicaemia virus.

In this work, we have firstly defined the appropriate infectious dosage for wt-VHSV and dNV-VHSV in order to establish the comparative transcriptomic profiling between them. At 48 h post-infection, the RNA transcripts from head kidney and spleen were analysed by RTqPCR to estimate the corresponding viral replication loads based on *N* transcript levels (Figure 1). The trout injected with 10^4^ pfu, 10^5^ pfu, and 35 × 10^6^ pfu of the wt-VHSV yielded the following *N* (±SD) values: 2.3 ± 1.1, 9.0 ± 4.1, and 348.8 ± 125, respectively (Figure 1). On the other hand, trout injected with 10^4^ pfu, 10^5^ pfu and 35 × 10^6^ pfu of the dNV-VHSV yielded the following *N* (±SD) values: 0.6 ± 0.2, 1.1 ± 0.4, and 19.3 ± 7.6, respectively (Figure 1). Wild-type-VHSV/dNV-VHSV ratio for *N* transcripts yielded an approximately 18-fold higher replication rate for the wt-VHSV when both viruses were applied at a dose of 35 × 10^6^ pfu/trout dose. However, this proportion was close to 1 when with the dose used was 10^5^ pfu for wt-VHSV and 35 × 10^6^ pfu for dNV-VHSV. For this reason, we considered it best to use the latter for the microarray study. Therefore, we compared the transcriptomic profile of trout injected with 10^5^ pfu of wt-VHSV (NV presence) (data deposited on GEO GSE37330) and trout injected with a 350-fold more infectious dose (35 × 10^6^ pfu) of dNV-VHSV (NV absence) (data deposited at GEO GSE43285).

### 2.2. Overview of the Expression Profiles Obtained

**Figure 2:**
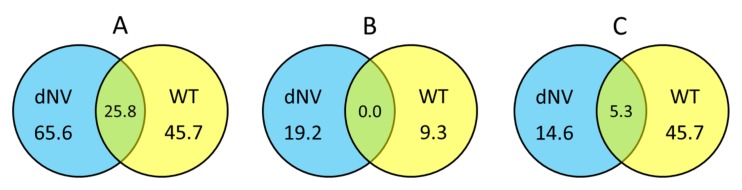
Venn diagrams showing the general transcriptomic relationships between trout injected with dNV-VHSV and wt-VHSV. Numbers indicate the percentage of upregulated (fold change, FC ≥ 1.5, (**A**), downregulated (FC ≤ −1.5, (**B**), and non-regulated (−1.5 < FC < 1.5, (**C**) genes, 48 h after injection with dNV-VHSV and wt-VHSV.

Normalised FC values of microarray datasets from wt-VHSV (10^5^ pfu/trout) and dNV-VHSV (35x10^6^ pfu/trout) were classified based on the groups defined for this study (see Section 1.5) and calculated the percentage of upregulated, downregulated and non-regulated genes. Figure 2A shows the upregulated genes between groups (wt-VHSV and dNV-VHSV) with fold changes (FC) ≥ 1.5 in Venn diagrams. The trout injected with dNV-VHSV showed the highest number of upregulated genes (65.6%), followed by those injected with wt-VHSV (45.7%). Probably, the upregulation increase in the dNV-VHSV injected trout was due to the absence of NV. In addition, dNV-VHSV and wt-VHSV shared 25.8% of the upregulated genes, which should be due to other viral proteins rather than NV.

Figure 2B displays Venn diagrams with downregulated genes (FC ≤ −1.5), which indicate that fish infected with dNV-VHSV downregulated more genes than fish infected with wt-VHSV (19.2% vs 9.3%, respectively). On the other hand, dNV-VHSV and wt-VHSV do not have any downregulated genes in common.

When the non-regulated gene transcript levels with folds −1.5 < FC < 1.5 (Figure 2C) were analysed by Venn diagrams, the trout infected with wt-VHSV had the highest number of non-regulated genes (45.7%), followed by dNV-VHSV (14.6%). The Venn diagram also showed that 5.3% of non-regulated genes were shared by wt-VHSV and dNV-VHSV.

### 2.3. dNV- and wt-VHSV Infection Effects on Trout Immune-Related Genes

A detailed study of the transcriptomic changes driven by wt-VHSV and dNV-VHSV at 48 hpi was conducted. A heatmap was generated with the FC of the genes grouped in the categories described in methods. In addition, individual genes were also correlated with the pathways (KEGG database) [32] in which they participate (Table 1).

#### 2.3.1. Cytosolic Sensors

**Figure 3:**
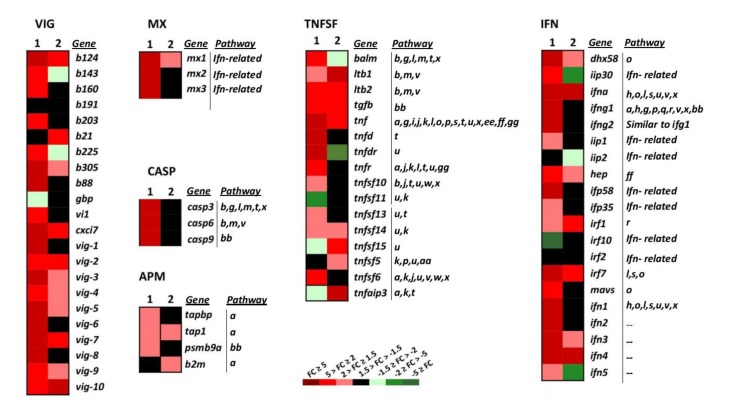
Heatmap showing the transcriptional expression fold changes (FCs) of the selected gene groups VHSV-induced (VIG), myxovirus resistance proteins (MX), caspases (CASP), tumour necrosis factor superfamily (TNFSF), interferon (IFN), and antigen-presenting machinery (APM) induced by dNV-VHSV and wt-VHSV in infected trout. *Gene* denotes names, and the *Pathway* column correlates with Table 1. --, unassigned pathway. Column 1, dNV-VHSV, each box corresponds to the average FC from four trout. Column 2, wt-VHSV, each box corresponds to the average FC from four trout. VIG group: b191 (c-lectin, AF483535), vig-1 (AF076620), vig-2 (AF290477), vig-3 (AF483529), vig-4 (AF483530), vig-5 (clone B17), vig-6 (clone B126), vig-7 (AF483527), vig-8 (clone B68), vig-9 (AF483533), vig-10 (AF483534), b203 (AF483538), b143 (AF483539), b225 (AF483540), b88 (AF483541), b160 (AF483545), b124 (AF483546), b305 (AF483542), cxci7 (VHSV induced protein 7 (vig7)), vi1 (VHSV induced protein 1), gbp (guanylate-binding protein GTPase, b21 (CD9, AF483544)). MX group: mx1 (myxovirus resistance 1), mx2 (myxovirus resistance 2), mx3 (myxovirus resistance 3). CASP group: casp3 (caspase 3), casp6 (caspase 6), casp9 (caspase 9). TNFSF group: balm (BAFF and APRIL-like molecule), ltb1 (lymphotoxin beta 1), ltb2 (lymphotoxin beta 2), tgfb (tumour growth factor beta), tnf (tumour necrosis factor alpha), tnfd (tumour necrosis factor decoy), tnfdr (tumour necrosis factor decoy receptor), tnfr (tumour necrosis factor receptor), tnfsf10 (tumour necrosis factor superfamily 10), tnfsf11 (tumour necrosis factor superfamily 11), tnfsf13 (tumour necrosis factor superfamily 13), tnfsf14 (tumour necrosis factor superfamily 14), tnfsf15 (tumour necrosis factor superfamily 15), tnfsf5 (tumour necrosis factor superfamily 5 (CD40)), tnfsf6 (tumour necrosis factor superfamily 6), tnfaip3 (tumour necrosis factor alpha-induced protein 3). IFN group: dhx58 (RIG-I-like receptor LGP2), iip30 (interferon gamma inducible protein 30), ifna (interferon alpha), ifng1 (interferon gamma 1), ifng2 (interferon gamma 2), iip1 (interferon inducible protein 1), iip2 (interferon inducible protein 2), hep (hepcidin), ifp58 (interferon-induced protein 58), ifp35 (interferon-induced protein 35), irf1 (interferon regulatory factor 1), irf10 (interferon regulatory factor 10), irf2 (interferon regulatory factor 2), irf7 (interferon regulatory factor 7), mavs (mitochondrial antiviral signalling protein), ifn1 (type 1 interferon 1), ifn2 (type 1 interferon 2), ifn3 (type 1 interferon 3), ifn4 (type 1 interferon 4), ifn5 (type 1 interferon 5). APM group: tapbp (tapasin (TAP binding protein)), tap1 (transporter associated with antigen processing 1), psmb9a (proteasome subunit type 9), b2m (beta-2 microglobulin).

**Figure 4:**
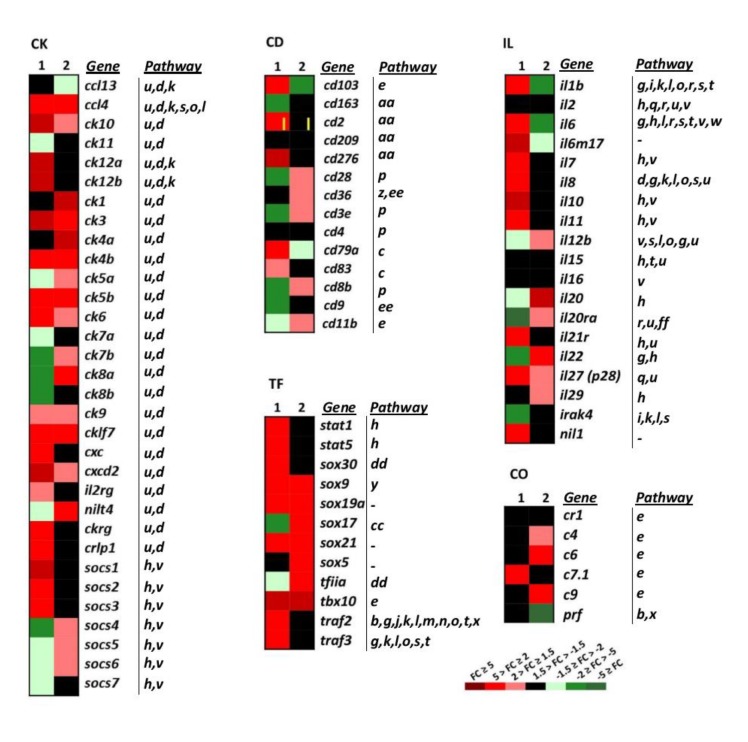
Heatmap showing the transcriptional expression fold changes (FCs) of the selected gene groups cytokines (CK), interleukins (IL), cluster of differentiation antigens (CD), complement (CO) and transcription factor (TF), induced by dNV-VHSV and wt-VHSV. *Gene* denotes names, and the *Pathway* column correlates with Table 1. --, unassigned pathway. Column 1, dNV-VHSV, each box corresponds to the average FC from four trout. Column2, wt-VHSV, each box corresponds to the average FC from four trout. CK group: ccl13 (cc-chemokine 13), ccl4 (cc-chemokine 4), ck10 (cc-chemokine 10), ck11 (cc-chemokine 11), ck12a (cc-chemokine 12a), ck12b (cc-chemokine 12b), ck1 (cc-chemokine 1), ck3 (cc-chemokine 3), ck4a (cc-chemokine 4a), ck4b (cc-chemokine 4b), ck5a (cc-chemokine 5a), ck5b (cc-chemokine 5b), ck6 (cc-chemokine 6), ck7a (cc-chemokine 7a), ck7b (cc-chemokine 7b), ck8a (cc-chemokine 8a), ck8b (cc-chemokine 8b), ck9 (cc-chemokine 9), cklf7 (chemokine-like factor superfamily member 7), cxc (α-chemokines), cxcd2 (cxc d2 chemokine), il2rg (il2 receptor gamma), nilt4 (novel immunoglobulin-like transcript 4 (FM200774.1)), ckrg (cytokine receptor gamma), crlp1 (chemokine receptor-like protein 1 (AJ620468.1)), socs1 (suppressor of cytokine signalling 1), socs2 (suppressor of cytokine signalling 2), socs3 (suppressor of cytokine signalling 3), socs4 (suppressor of cytokine signalling 4), socs5 (suppressor of cytokine signalling 5), socs6 (suppressor of cytokine signalling 6), socs7 (suppressor of cytokine signalling 7). IL group (r means receptor): il1b, il2, il6, il6m17, il7, il8, il10, il12b, il15, il16, il20, il20ra, il21r, il22, il27 (p28 subunit), il29, Irak4 (interleukin-1 receptor-associated kinase 4), nil1 (novel il-1 cytokine family member). CD group: cd103, cd163, cd2, cd209, cd276, cd28, cd36, cd3e (epsilon), cd4, d79a, cd83, cd8b (beta), cd9. CO group: cr1 (complement receptor type 1), c4 (complement component 4), c6 (complement component 6), c7.1 (complement component 7-1), c9 (complement component 9), prf (perforin). TF group: stat1 (signal transducer and activator of transcription 1), stat5 (signal transducer and activator of transcription 5), sox30 (SRY-related HMG box 30 gene family), sox9 (SRY-related HMG box 9 gene family), sox19a (SRY-related HMG box 19a gene family), sox17 (SRY-related HMG box 17 gene family), sox21 (SRY-related HMG box 21 gene family), sox5 (SRY-related HMG box 5 gene family), tfiia (transcription factor IIA), tbx10 (T-box 10 gene).

Upon infection, viruses are recognised by host receptors and cytosolic sensors that activate mechanisms (signalling molecules) involved in diverse cellular processes such as the antiviral immune response. The cytosolic sensors studied belong to different gene groups and signalling pathways (Figure 3 and Figure 4, and Table 1, RIG-I-like, and NOD-like signalling). Among the genes involved in these pathways, *tnf*, *ifna*, *irf7*, and *dhx58* and *mavs* (Figure 3, IFN and TNFSF) were upregulated by wt-VHSV and slightly more by dNV-VHSV. *Mavs*, another important cytosolic sensor, was upregulated by dNV-VHSV but non-regulated by wt-VHSV. This fact could be due to the NV expression by wt-VHSV and might indicate that *mavs* would be more functional for starting an appropriate immune response against dNV-VSHV as vaccine virus. Other important signalling molecules are *traf2* and *traf3* (Figure 3, TNFSF), and they are also upregulated by dNV-VHSV and non-regulated by wt-VHSV. These findings also support that dNV-VHSV activates better than wt-VHSV at these stages.

#### 2.3.2. IFN System

We studied different genes belonging to the IFN system and IFN-related group of genes that belong to different signalling pathways (Figure 3, IFN pathways, *n* = 14). Regarding the IFN group of genes (IFN, *n* = 20), wt-VHSV induced the upregulation of the lowest number of genes (35%) compared to dNV-VHSV (85%) (Figure 3, IFN). This fact might be due to an inhibitory effect driven by NV after its expression from wt-VHSV. Among the downregulated and non-regulated (basal) genes, we found *iip30*, *ifng1*, *ifng2*, *iip1*, *iip2*, *ifp58*, *ifp35*, *mavs*, *ifn1*, *ifn2*, and *ifn5* (Figure 3, IFN). On the other hand, the expressions of *dhx58*, *hep*, *ifn3*, *ifna*, *irf1*, *irf7*, and *ifn4* were upregulated by wt-VHSV. Thus, some of them (i.e., *dhx58*, *hep*, *irf7*, *ifn3*) were even more upregulated by dNV-VHSV, probably due to the lack of the NV protein. The NV protein expressed by wt-VHSV induced the non-regulation of *mx2* and *mx3*, whereas *mx1* was slightly upregulated. The results also showed that all *mx* genes were upregulated by dNV-VHSV (Figure 3, MX). Mx proteins are implicated in the antiviral interferon-mediated response. In summary, in contrast to wt-VHSV, dNV-VHSV improved the antiviral immune response based on interferons (and related molecules), which would support its use as a potential live vaccine.

#### 2.3.3. TNF Superfamily and Caspases

This gene group is comprised of molecules with diverse functionality that participate in several signalling pathways (*n* = 20, Figure 3 TNFSF, and Table 1). Most of the 16 TNF superfamily genes (*tnfsf*) studied here were upregulated (75%) by dNV-VHSV, whereas wt-VHSV induced a lower upregulation (50%) of these genes (Figure 3, TNFSF). The *tnfsf* genes upregulated by dNV-VHSV but downregulated or non-regulated by wt-VHSV were *balm*, *tnfd* (decoy), *tnfdr* (decoy receptor), *tnfr*, *tnfsf10*, *tnfsf13*, *tnfsf14,* and *tnfsf6* (Figure 3, TNFSF), which participate in several multipaths (Figure 3, TNFSF, Table 1). The *balm* gene is closely related to *tnfsf13b* (BAFF) and *tnfsf13* (APRIL) genes, and seems to be unique to teleost [33]. The *balm* gene has a constitutive expression in adult trout, mainly in the spleen, lymphocytes, posterior kidney, and anterior kidney and, therefore, *balm* has been assigned an immunological role [33]. The *tnfsf6* (FAS ligand, FASL) and *tnfsf13* (APRIL) genes modulate ligand-induced apoptosis [34]. In addition, *tnfsf10* (or TRAIL) is an inductor of apoptosis acting through *casp3* and *casp8*. Among the upregulated genes induced by wt- and dNV-VHSV injection were *tnfsf14* (stimulator of apoptosis), *tnf* (most important inducer of systemic inflammation), and *ltb1/ltb2* (involved in proliferation, differentiation, survival, and growth). Another member of TNFSF gene group is *tnfaip3* (A20 protein), which is upregulated in trout infected with wt-VHSV and downregulated in those infected with dNV-VHSV. Previous studies showed that A20 inhibited NF-kappa B and apoptosis [35,36] and our results support that dNV-VHSV would promote NF-kappa B signalling in order to set up a successful immune response.

TNFs have a role as ‘double-edged swords’ in cellular proliferation, survival, differentiation or apoptosis. Ligands such as APRIL (*tnfsf13* gene), LIGHT (*tnfsf1*4 gene), RANKL (*tnfsf11* gene), LT-β (*ltb1*, *ltb2* genes), and CD40L (*tnfsf5* gene) bind to receptors with a TRAF-interacting motifs (TIM) domain, leading to the recruitment of TRAF molecules, and the activation of multiple signal transduction pathways such as NF-kappa B, Jun N-terminal kinase (JNK), p38, extracellular signal regulated kinase (ERK), and phosphoinositide-3 kinase (PI3K). On the other hand, ligands such as TNF-α (*tnf* gene), TRAIL (*tnfsf10* gene), FASL (*tnfsf6* gene), and decoy receptor have a dead domain (DD), which ultimately activates apoptosis through caspases. In summary, most of the *tnfsf* genes were upregulated by dNV-VHSV, giving rise to both activation signalling (NF-Kappa B, JNK, etc) and apoptosis signalling pathways.

Regarding the caspase group (Figure 3, CASP, and Table 1), the effector (*casp3*, *casp6*) and initiator (*casp9*) caspase genes were highly upregulated in the dNV-VHSV infected group while maintained at normal transcription levels in the wt-VHSV infected group. These results suggest that NV expressed by wt-VHSV impairs the upregulation and, consequently, the activation of apoptosis at 48 h post-infection (Figure 3, CASP). Moreover, the upregulation of the *casp* genes by dNV-VHSV promotes the activation of different pathways in which they participate, supporting an appropriate immune response developed by dNV-VHSV.

#### 2.3.4. Antigen Presentation

Among the antigen presenting machinery (APM, *n* = 4) genes studied, TAPASIN (*tapbp*) and proteasome subunit (*psmb9a*) were non-regulated genes, whereas *tap1* and *b2m* were upregulated in wt-VHSV (Figure 3, APM). The APM genes belong to innate and adaptive immune responses. They act within the proteasome for peptide generation (*psmb9a*) and the transport of peptides (*tapbp*, *tap1*) into the endoplasmic reticulum. MHC (Major histocompatibility complex) class I molecules (together with *b2m*) bind these antigenic peptides to present them to CD8+ T-lymphocytes. Other genes belonging to TNFSF and IFN groups are also implicated in the antigen presentation signalling pathway (Figure 3). Overall, the results indicated that dNV-VHSV favoured the antigen processing and presentation in relation to wt-VHSV.

#### 2.3.5. Cluster of Differentiation: B-Cell, T-Cell, and Cell-to-Cell Interactions

Cluster of differentiation genes (CD) conform a functional heterogeneous group of genes that have been involved in cell adhesion, B-cell receptor signalling, T-cell receptor signalling, complement and coagulation cascades, and hematopoietic markers (Figure 4 CD, and Table 1). In this study, 14 CD genes have been analysed. Downregulated and non-regulated CD genes (CD, *n* = 5 genes, Figure 4 CD) were found in the wt-VHSV dataset. The downregulated genes by wt-VHSV were *cd103* (a marker of dendritic cells) [37] and *cd79a* (associated with membrane-bound immunoglobulin in B-cells). On the other hand, the non-regulated genes in wt-VHSV were *cd2* (implicated in the adhesion T cell-APC through the *CD58* protein), *cd276* (participating in the regulation of the T-cell-mediated immune response), *cd83* (involved in the regulation of antigen presentation) [38], and *cd163* (exclusively expressed in monocytes and macrophages in humans), which was downregulated by dNV-VHSV. Some of the CD markers were found upregulated by dNV-VHSV (*cd103*, *cd2*, c*d276*, *cd279*, *cd83*) and others upregulated by wt-VHSV (*cd28*, *cd36*, *cd3e*, *cd83*, *cd11*). This fact might indicate that 48 hpi is a too short time to observe adaptive cellular responses against VHSV.

#### 2.3.6. Cytokines: Chemokines and Interleukins

Among the chemoattractant cytokines or chemokines genes (CK, *n = 32*), 53% were upregulated by wt-VHSV, whereas dNV-VHSV induced the upregulation by 50%. The chemokines showing downregulated or non-regulated fold changes by wt-VHSV were *ccl13*, *ck11*, *ck12a*, *ck12b*, *ck8b*, *cxc*, *ckrg*, *crlp1*, suppressor of cytokine 1 (*socs1*), *socs2*, *socs3*, and *socs7* (Figure 4, CK). In addition, dNV-VHSV induced the downregulation or non-regulation of the chemokines *ccl13* (basal), *ck1*, *ck4a*, *ck5a*, *ck7a*, *ck7b*, *ck8a*, *ck11*, *nilt4*, *socs4*, and *socs5*. Chemokines have different roles in the coordination of the immune response and may promote the activation or inhibition of different pathways (Figure 4 IL, Table 1), and for the most of them their function is unknown on the basis of viral infections.

Previous studies in rainbow trout have shown that recombinant CK1 has an attractant effect for blood leukocytes) [39]. In addition, CK6 is a chemoattractant for mature macrophages from the RTS11 rainbow trout monocyte-macrophage cell line and may also induce interleukin 8 (IL-8), inducible nitric oxide synthase (iNOS), and the CD-18 integrin in these cells, revealing additional immunomodulatory effects [40]. The capacity of trout recombinant CK12 to attract splenocytes has also been reported, establishing that IgM + B cells were one of the target cells recruited [41]. In the present study, CK1, CK6 and CK12 are upregulated by dNV-VHSV in relation to wt-VHSV. Regarding the interleukin genes group (IL, *n* = 20), the transcriptomic profile obtained after wt-VHSV injection was different from that of dNV-VHSV. Wild-type VHSV downregulated important pleiotropic pro- and anti-inflammatory interleukins such as *il1b, il6,* and its related *il6m17* (Figure 4, IL). On the contrary, interleukins *il1*, *il6*, *il6m7, il7*, *il8*, *il11*, *il21r*, *il27* and *nil1* were upregulated by dNV-VHSV. These interleukins have a key role in immune pathways (Figure 4 IL, Table 1) and their upregulation are required for an effective immune response followed vaccination, suggesting that dNV-VHSV could be an effective attenuated live vaccine.

#### 2.3.7. General Transcription Factors

This group of genes (TF, *n* = 10 selected genes, Figure 4) are implicated in important cellular processes and pathways: Jak–Stat signalling (*stat1*, *stat5*), general transcription factors and regulatory elements (*sox* genes), and multipath genes previously mentioned (*traf2*, *traf3*). In the dNV-VHSV-injected trout, all the TF selected genes were upregulated except for *sox5* (non-regulated) and *tfiia* (downregulated). In wt-VHSV, all the genes studied were upregulated except for *traf2*, *traf3*, *sox30*, *stat5* and *stat1*, which were non-upregulated. These data reflected that Jak–Stat signalling pathway is upregulated by dNV-VHSV, which in turn promoted interferon responses leading to an improved antiviral stage.

#### 2.3.8. Complement and VIG Genes

Among complement genes studied (CO, *n* = 6, Figure 4), the trout injected with dNV-VHSV maintained most of the genes non-regulated and only one was upregulated. It is interesting to note that C9 was upregulated by wt-VHSV, whereas perforin (*prf*) was downregulated by the same virus.

VHSV-induced genes (VIG, *n* = 22) were firstly identified by subtractive hybridisation performed in a previous work [42]. We found that NV from wt-VHSV inhibited the upregulation of 50% of all *vig* genes (*b143*, *b160*, *b191*, *b203*, *b225*, *b88*, *gbp*, *vi1*, *vig-1*, *vig-6*, *vig-8*) while these genes were upregulated in dNV-VHSV, except for *b191* and *gbp* (Figure 3, VIG). Viperin (*vig1*) is expressed by mitochondria and is an IFN-inducible protein that inhibits the replication of a variety of viruses [43]. In addition, *vig-2* [44], *vig-3*, *vig-4*, *vig5*, and probably *vig-6* are also induced by interferon [42]. On the other hand, *vig-7*, *vig-8*, *vig-9* have chemoattractant function, *vig-9* also has an apoptotic function, and *vig-10* is related to apoptosis and transcription repression [42].

The present study is the first one describing the trout transcriptomic profile driven by dNV-VHSV. There are scarce studies regarding whole gene effects upon VHSV infection in trout, the most relevant to our study is the characterization of the RNA microarray profile in olive flounder liver after VHSV infection by immersion [20]. Regarding the role of immune-related genes, we found that both wt-VHSV and dNV-VHSV induced an upregulation of hepcidin, (a regulator of the iron metabolism that is implicated in inflammatory processes) in trout similar to the results observed in VHSV infected olive flounder. However, the *irf2* gene was found non-regulated in the wt-VHSV injected trout, which differs from the upregulation found in olive flounder .The differences found between trout and olive flounder could be due to the expression profile of immune response genes in spleen/head kidney, which could be slightly different from those in liver.

Another recent study determined the miRNA expression profile after VHSV infection. Among the immunity-associated target genes of 63 differentially expressed miRNA after VHSV infection of olive flounder, the authors described IL (*il1b*, *il8*, *il10*), *mx*, interferon regulatory factors (*irf3*, *irf5*, *irf7*), TNF (*tnfsf*), and heat shock proteins (*hsp10*, *hsp60*, *hsp70*, *hsp90*) [45]. For instance, *mx* mRNA was regulated by one miRNA (pol-miR-1388-5p) only, for which the highest expression was at 0 hpi and the lowest at 72 hpi. At 48 hpi, the *mx* expression was not modulated by this miRNA. Our data in trout showed a non-regulation of *mx* at 48h after wt-VSHV injection, being coincident with those of miRNA in olive flounder. Maybe in the future, a correlation between miRNA and mRNA expression levels could be established in infected VHSV fish, as it has been done for some miRNAs in primary human osteoblasts from healthy individuals [46].

### 2.4. Modulated Pathways in dNV- and wt-VHSV-Injected Trout using GSEA

**Figure 5:**
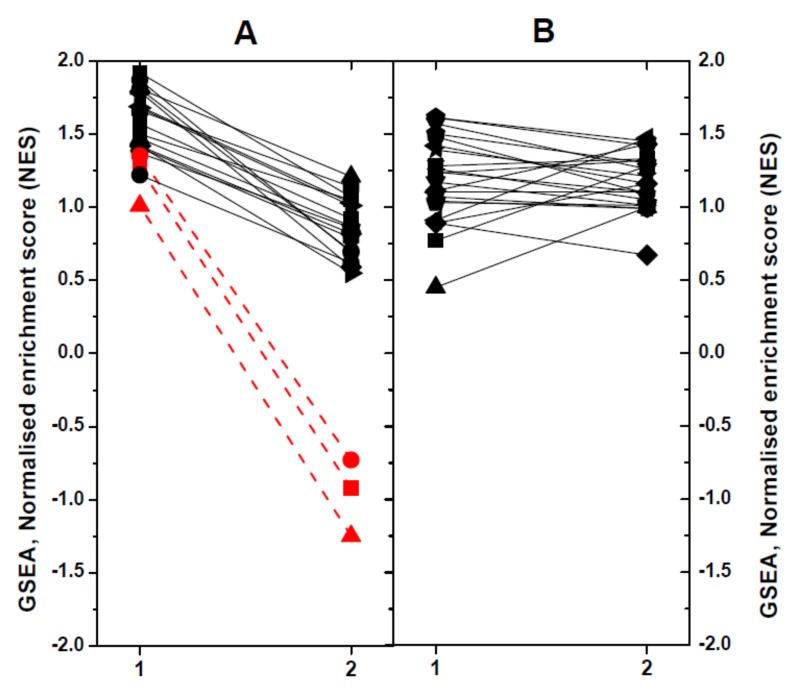
Comparison of significant normalised enrichment scores (NESs) of rainbow trout gene-sets (GSs) by Gene-Set Enrichment Analysis (GSEA). X axis: 1, dNV-VHSV. 2, wt-VHSV. Trout GSs were obtained from the KEGG (-K) and WIKI (-W) pathway databases as described before [25]. For comparative purposes, the NESs calculated by GSEA were represented in a diagram by linking the results obtained with lines. For better clarity, the results were not individually identified by GS. (**A**) GS which show upregulation in dNV-VHSV in relation to wt-VHSV: Toll-like receptor wikipathway-W, Hepatitis C-K, Toll-like receptor signalling pathway-W, RIG-I-like receptor signalling-K, Influenza A-K, Type II interferon signalling (IFNG)-W, Measles-K, NF-kappa B signalling pathway-K, TNFa NF-kappa B signalling-W, Herpes simplex infection-K, Epithelial cell *Helicobacter pylori*-K, T cell receptor signalling pathway-K, Cytosolic DNA sensing pathway-K, Natural killer cell mediated cytotoxicity-K, Interleukin 5-W, Cytokine inflammatory response pathway-W. The most upregulated pathways by dNV-VHSV in relation to wt-VHSV were: Red circle, Autoimmune thyroid disease-K; Red square, Regulation of autophagy-K; and Red triangle, Protein processing in endoplasmic reticulum-K. (**B**) GS which showed no regulation in relation to wt-VHSV: Interferon type I-W, Interferon alpha beta signalling-W, Apoptosis modulation by HSP70-W, B-cell receptor signalling pathway-W, EGFR1 signalling pathway-W, MAPK signalling pathway-K, Interleukin 6-W, Ubiquitin mediated proteolysis-K, TSH signalling pathway-W, Antigen processing and presentation-K, MAPK signalling pathway-W, Interleukin 2-W, HTLV-K, androgen receptor signalling-W, TP53 network signalling-W, AHR pathway-W, Interleukin 3-W, Hematopoietic cell lineages-K, T-cell receptor pathway-W, JAK–STAT signalling pathway-K, PI3K–AKT signalling pathway-K, FGF signalling pathway-W.

To define the impact of dNV-VHSV or wt-VHSV when targeting different pathways or gene sets (GS) in head kidney/spleen, we used the Gene-Set Enrichment Analysis (GSEA) program. Briefly, 51 trout GS collection previously defined [25] were used to obtain normalised enrichment scores (NES). The GS were then classified according to their NES values, as follows: (1) upregulation in dNV-VHSV in relation to wt-VHSV (*n* = 19), and (2) no regulation in dNV-VHSV in relation to wt-VHSV (Figure 5). Among the upregulated GS in dNV-VHSV in relation to wt-VHSV (Figure 5A), higher differences were found in those corresponding to “Protein processing in endoplasmic reticulum”, “Regulation of autophagy”, and “Autoimmune thyroid disease” (> 2 to 4-fold) (Figure 5A, red lines and symbols). Other GSs which showed lower improvement of upregulations with dNV-VHSV were those implicated in anti-viral interferon networks (“Toll-like receptor signalling pathway”, “Toll-like receptor wikipathway”, “Type II interferon signalling (IFNG)”), inflammation (“TNFa NF-kappa B signalling”, “Cytokine inflammatory response pathway”), recognition of nucleic acids (“Cytosolic DNA sensing pathway”, “RIG-I-like receptor signalling”), viral- and bacterial-caused diseases (“Hepatitis C”, “Influenza A”, “Measles”, “Herpes simplex infection”, “Epithelial cell *Helicobacter pylori*”), and others (“NF-kappa B signalling pathway”, “T cell receptor signalling pathway”, “Natural killer cell mediated cytotoxicity-K”, “Interleukin 5”). The remaining GSs showed no differences between dNV-VHSV and wt-VHSV (Figure 5B). The “Type II IFN signalling (IFNG)” pathway was one of the top enriched GS found in VHSV survivor zebrafish [47], indicating that both species share similar mechanisms to fight VHSV. Other improved pathways in dNV-VHSV such as “Toll-like receptor signalling”, “RIG-I-like receptor signalling”, “Natural killer cell-mediated cytotoxicity”, “Hepatitis C”, and “Influenza A and Measles” were among the most targeted pathways in SVCV zebrafish infections [48]. Finally, “NF-kappa B signalling pathway”, “Toll-like receptor wikipathway”, “Natural killer cell mediated cytotoxicity”, “RIG-I-like receptor signalling”, “Autoimmune thyroid disease”, “Influenza A”, and “Herpes simplex infection” were also modulated in trout when injected with thyroid hormone analogues [25]. All the above-mentioned pathways participated in generating resistance to fish viral infections and underlined the importance that their upregulation by dNV-VHSV might have in case this defective virus is used as a potential attenuated live vaccine.

## 3. Conclusions

The results presented in this study support the hypothesis that dNV-VHSV can be considered an attenuated virus and a potential live vaccine, based on the fact that many critical host gene pathways are activated upon infection. On the contrary, NV expressed at first stages of infection by wt-VHSV modulates the expression levels of interferons, VIG, chemokines, CD, transcription factors, and other immune-related genes, leading to an immune unresponsiveness state that interferes with the early innate immune response. Importantly, this work opens new avenues for the use of NV-deleted novirhabdoviruses as a tool to study the regulation of immune pathways in other teleost fish.

## Figures and Tables

**Table 1:** Pathways related to modulated genes by WT-VHSV and dNV-VHSV. The pathways were obtained from the KEGG database [32]. See also Figure 3 and Figure 4 for correlation with the genes.

(a) Antigen processing and presentation	(q) Th1 and Th2 cell differentiation	
(b) Apoptosis	(r) Th17 cell differentiation	
(c) B-cell receptor signalling	(s) TLR (Toll-like receptor) signalling	
(d) Chemokine signalling	(t) TNF (Tumour necrosis factor) signalling	
(e) Complement and coagulation cascades	(u) Cytokine-cytokine receptor interaction	
(f) ErbB (Erb-B2 Receptor Tyrosine Kinase 2) signalling		
	(v) PI3-Akt (phosphatidylinositol 3-kinase and Protein kinase B ) signalling	
	(w) FoxO (Forkhead box O)signalling	
(g) IL-17 signalling		
	(x) Natural killer cell-mediated cytotoxicity	
(h) Jak–Stat signalling (Janus kinase-Signal transducer and activator of transcription)	(y) cAMP (Cyclic adenosine 3′,5′-monophosphate) signalling	
(i) MAPK (Mitogen-Activated Protein Kinase) signalling	(z) AMPK (AMP-activated protein kinase) signalling	
(j) Necroptosis signalling	(aa) Cell adhesion molecules	
(k) NF-kappa B signalling (Nuclear Factor kappa-light-chain-enhancer of activated B cells)	(bb) Proteasome	
	(cc) Wnt (wingless-type MMTV integration site family) signalling	
(l) NOD-like (nucleotide-binding oligomerization domain-like) receptor signalling	(dd) General transcription factors	
	(ee) Hematopoietic cell lineage	
	(ff) TGFb (Transforming growth factor beta) signalling	
(m) p53 signalling	(gg) mTOR (mammalian target of rapamycin) signalling	
(n) Protein processing in endoplasmic reticulum		
(o) RIG-I-like receptor signalling		
(p) T-cell receptor signalling		
